# Recent Advances in Communications and Networking

**DOI:** 10.1155/2014/376260

**Published:** 2014-12-18

**Authors:** Zhongmei Zhou, Yuxin Mao, Jaime Lloret, Xiaoxuan Meng, Jingjing Zhou

**Affiliations:** ^1^Department of Computer Science and Engineering, Minnan Normal University, Zhangzhou 363000, China; ^2^School of Computer and Information Engineering, Zhejiang Gongshang University, Hangzhou 310018, China; ^3^Department of Communications, Universidad Politecnica de Valencia, 46730 Valencia, Spain; ^4^VMware, 3401 Hillview Avenue, Palo Alto, CA 94304, USA; ^5^School of Information and Electronic Engineering, Zhejiang Gongshang University, Hangzhou 310018, China

The emerging communication and networking technologies and the way in which these are being integrated into the human, industrial, and social framework have made it evident that there are a number of related technical and socioeconomic areas whose understanding is still less than satisfactory and in which long-term research is needed. Meanwhile, a number of emerging concepts like cloud computing, Internet of Things, web 3.0, and green radio have been proposed in communication and networking. The research on emerging communication and networking technologies is considered as a global research challenge.

Therefore, we just hold this special issue for researchers, engineers, and practitioners to share their work on recent advances in communications and networking. The special issue focuses on the hottest and cutting-edge topics in communications and networking.

After peer-review, more than 30 papers from different affiliations and countries were accepted and published in this special issue. A feature of this special issue is that we have addressed the importance of wireless networking and accepted a number of papers related to this topic. We think that wireless networking will become more and more popular in both research and industry. Mobile network or MANET has been a hot topic during these years. Therefore, we have accepted a number of papers related to this topic in this special issue. As a special kind of MANET, VANET has also become a popular research topic recently. In this special issue, several papers are just talking about VANET, vehicular network, V2V, and urban traffic network. As an underlying technology for Internet of Things, wireless sensor network has been studied a lot. Many research efforts have been proposed in this field. We have also addressed some new research in this special issue.

The rest of the papers in the special issue have talked about a wide range of emerging topics like cloud computing, social networks, satellite networks, microgrid, network security, and so forth.

We hope that readers of this journal, especially those from the communications subject area, will find in this special issue not only the new ideas, cutting-edge information, new technologies, and applications of communication and networking, but also a special emphasis on how to solve various emerging problems.

*Zhongmei Zhou* *Zhongmei Zhou* *Yuxin Mao* *Yuxin Mao* *Jaime Lloret* *Jaime Lloret* *Xiaoxuan Meng* *Xiaoxuan Meng* *Jingjing Zhou* *Jingjing Zhou*

